# Delineating the glycoproteome of elongating cotton fiber cells

**DOI:** 10.1016/j.dib.2015.10.015

**Published:** 2015-10-26

**Authors:** Saravanan Kumar, Pankaj Pandey, Krishan Kumar, Vijayalakshmi Rajamani, Kethireddy Venkata Padmalatha, Gurusamy Dhandapani, Mogilicherla Kanakachari, Sadhu Leelavathi, Polumetla Ananda Kumar, Vanga Siva Reddy

**Affiliations:** a Plant Transformation Group, International Centre for Genetic Engineering & Biotechnology (ICGEB), New Delhi, India; b National Research Centre on Plant Biotechnology (NRCPB), IARI, New Delhi, India

## Abstract

The data presented here delineates the glycoproteome component in the elongating cotton fiber cells attained using complementary proteomic approaches followed by protein and N-linked glycosylation site identification (Kumar et al., 2013) [1]. Utilizing species specific protein sequence databases in proteomic approaches often leads to additional information that may not be obtained using cross-species databases. In this context we have reanalyzed our glycoproteome dataset with the *Gossypium arboreum*, *Gossypium raimondii* (version 2.0) and *Gossypium hirsutum* protein databases that has led to the identification of 21 N-linked glycosylation sites and 18 unique glycoproteins that were not reported in our previous study. The 1D PAGE and solution based glycoprotein identification data is publicly available at the ProteomeXchange Consortium via the PRIDE partner repository (Vizcaíno et al., 2013) [2] using the dataset identifier PXD000178 and the 2D PAGE based protein identification and glycopeptide approach based N-linked glycosylation site identification data is available at the ProteomeXchange Consortium via the PRIDE partner repository (Vizcaíno et al., 2013) [2] using the dataset identifier PXD002849.

**Specifications Table**

**Table:** 

Subject area	Biology, Chemistry
More specific subject area	Glycoproteomics
Type of data	Table, text file, figure
How data was acquired	Mass Spectrometer, data acquired using Nano-LC-MALDI TOF/TOF (Ultraflex III, Bruker Daltonics)
Data format	Raw and Analyzed data
Experimental factors	Cotton fiber glycoproteins were enriched using Concanavalin A based lectin affinity chromatography followed by protein identification by Mass spectrometry using five independent protein databases.
Experimental features	1D SDS-PAGE, 2D SDS-PAGE, Gel free approach and Glycopeptide enrichment strategies were employed to elucidate the glycoproteome
Data source location	Plant Transformation Group, International Centre for Genetic Engineering and Biotechnology, New Delhi, India
Data accessibility	The data is accessible via this article, via the related research article [1], and at the ProteomeXchange Consortium via the PRIDE partner repository [2] using the dataset identifier PXD000178 and PXD002849. (http://proteomecentral.proteomexchange.org)

**Value of the data**

• The present data provides valuable insights about the glycoproteins present in the elongating cotton fiber cells identified using *Gossypium* species specific protein sequence databases.
• 127 N-linked glycosylation sites, from 81 unique glycoproteins including 21 N-linked glycosylation sites corresponding to 17 unique glycoproteins are exclusively reported in the current study.
• Our analyses using five independent protein databases show that *Gossypium hirsutum* harbors protein sequences from its parental contributors *Gossypium arboreum* and *Gossypium raimondii.*
• The elucidated glycoproteome composition indirectly provides clues about parental genome contribution and unicellular compartmental requirement behind single cell development.

## 1 Data

**Fig. 1:**
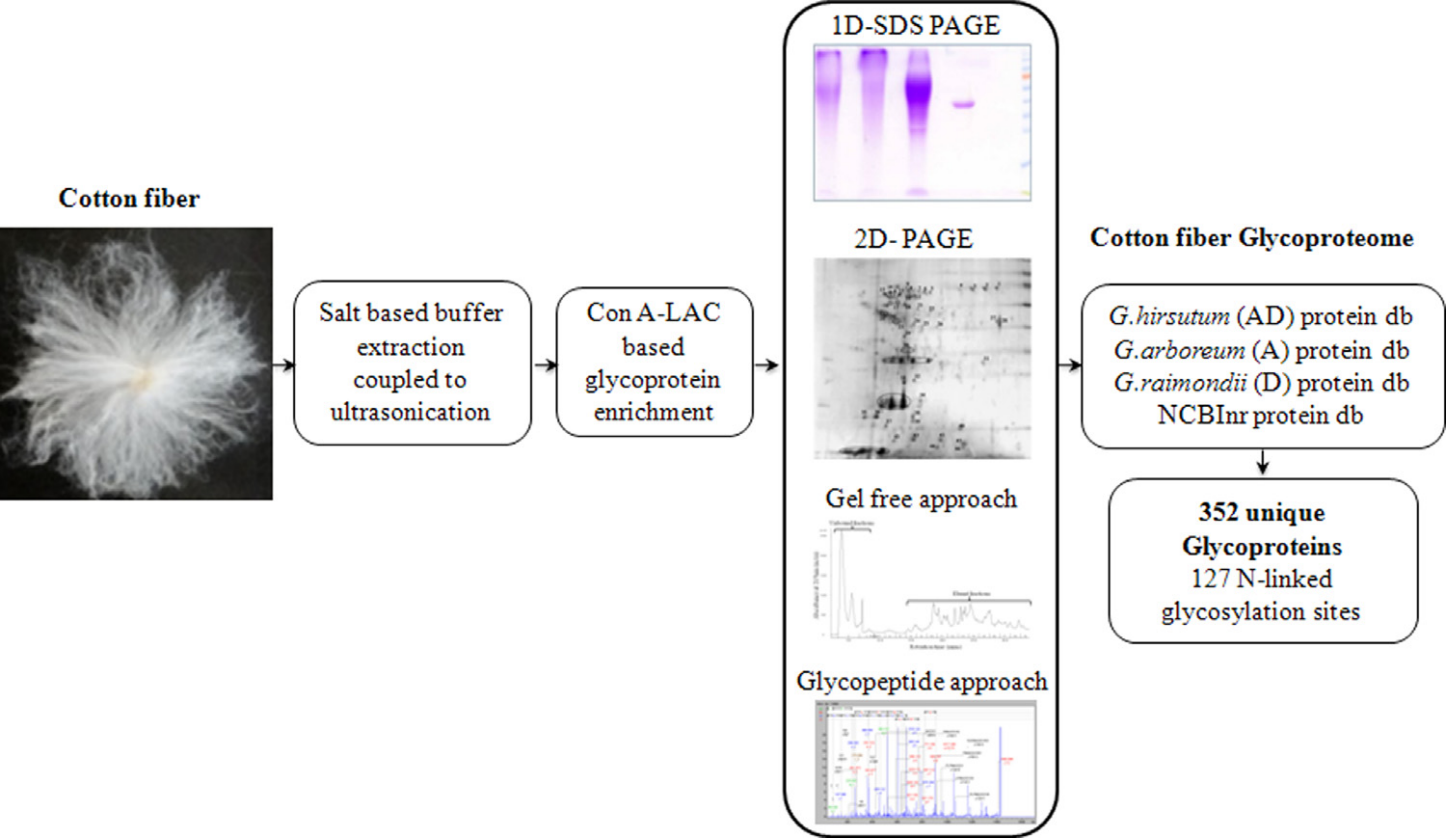
Diagrammatic representation of the workflow employed to elucidate cotton fiber glycoproteome.

Concanavalin A (Con A) based lectin affinity chromatography was employed to enrich glycoproteins isolated from elongating cotton fiber (Fig. 1). Four different proteomic approaches followed by five independent database search strategies were applied to identify the glycoproteins using Nano-LC-MALDI TOF/TOF. Our data revealed 352 unique proteins including 305 proteins (>86%) with potential N-linked glycosylation sites [1] (Table 1, Supplementary Table 1). Glycopeptide based enrichment approach revealed 127 N-linked glycosylation sites, from 81 unique glycoproteins (Table 1, Supplementary Table 2).

## 2 Experimental design

The role of glycosylated proteins in the structural and regulatory aspects of cotton fiber development is yet to be explored. In this context, we have optimized a salt based extraction coupled to ultrasonication procedure followed by lectin affinity chromatography based enrichment of cotton fiber glycoproteins (Fig. 1). In our previous study [1], protein identities were attained using the publicly available NCBInr and partially sequenced Cotton D genome (*Gossypium raimondii*) derived protein sequence databases as the complete genome sequence of *Gossypium hirsutum* and its parental species was not available at that time. Protein identities and posttranslational modification sites obtained using cross-species and partially sequenced protein databases are often incomplete. In this context, we have reanalyzed the dataset using the protein sequences from *G. hirsutum* (AD), *G. arboreum* (A) and *G. raimondii* (D, version 2) to explore additional protein identities and N-linked glycosylation sites that were not reported in our previous study [1].

## 3 Methods

### 3.1 Plant materials

Cotton plants (*G. hirsutum* cv. Coker 310) were grown in climate controlled green house. Cotton bolls were collected from plants during elongation stages (5–15 dpa) and fibers were carefully removed from the ovule, frozen immediately in liquid nitrogen and stored at −70 °C until further use.

### 3.2 Protein extraction

In order to isolate maximum amount of proteins from cotton fibers that is compatible with the downstream glycoprotein enrichment procedures, we have optimized a salt based buffer extraction followed by ultrasonication approach. Briefly, cotton fibers were made into fine powder and were suspended in extraction buffer containing 25 mM Tris (pH 7.5), 0.2 M CaCl_2_, 0.5 M NaCl, 20 mM β-mercaptoethanol (β-Me), 1X Proteinase inhibitor cocktail (Roche). The buffer extract was left under constant shaking for 2 h followed by intermittent vortexing at 4 °C. Ultrasonication of the suspended extract was performed at 35% amplitude for 10 min in ice cold condition. Sample extract was then centrifuged for 20 min at 10,000*g* and the supernatant was transferred into fresh centrifuge tubes. Three volumes of extraction buffer were again added to the pellet fraction and the extraction steps were repeated. The supernatants were pooled, filtered and dialyzed overnight. All the above mentioned steps were performed at 4 °C with three independent sample replicates. Dialyzed samples were frozen and lyophilized prior to use.

### 3.3 Glycoprotein capture by lectin affinity chromatography

Lyophilized crude protein extract was dissolved in binding buffer containing 20 mM Tris (pH 7.5), 0.5 M NaCl, 1 mM CaCl_2_,1 mM MnCl_2_, 1 mM MgCl_2_ and subjected to Concanavalin A (Con A) lectin affinity chromatography (LAC) in a manually packed column as described by Catala et al. [3]. In order to achieve maximum yield, glycoproteins bound to the lectin affinity column was eluted in three consecutive steps each with 3 column volumes (CVs) of binding buffer containing 0.5 M methyl α-d mannopyranoside (step I) followed by 1 M methyl α-d mannopyranoside (step II) and 1 M glucose (step III) respectively (Please see Fig. 1C in [1]). Eluant fractions were pooled, buffer exchanged and concentrated with buffer containing 20 mM Tris (pH 7.5) using Amicon 10 KDa (MWCO) centrifugal filters (Vivascience).

### 3.4 1D and 2D SDS PAGE

Around 50 μg of the protein samples enriched using LAC was subjected to 12% linear SDS-PAGE separation [4] in replicates. The gels were either stained with Coomassie, Periodic Acid Schiff (PAS) or β-glucosyl yariv stain to visualize the protein, glycoprotein or arabinogalactan pattern respectively. Around 100 μg of the CON-A enriched protein sample was subjected to two dimensional gel electrophoresis (2D SDS PAGE) using non-linear and linear immobilized pH gradient IPG strips. Briefly, for the first-dimensional separation, the sample was loaded onto a 13 cm IPG non-linear (pI 3–10) and linear (pI 4–7) strips (Amersham biosciences) and isoelectric focusing (IEF) was performed according to the manufacturer׳s instructions. Strips were then equilibrated and second-dimensional separation was carried out on 12% SDS polyacrylamide gel (13 cm, 1.5 mm). Gels were stained with silver staining procedure to visualize the spots and stored in 1% acetic acid at 4 °C until further use.

### 3.5 Gel phase digestion and gel free (solution phase) digestion

Glycoprotein samples resolved in 12% 1D PAGE gels were excised into 0.5 mm gel slices (18 slices) from high to low molecular weight region. Bands from 1D PAGE and spots from 2D PAGE containing the protein of interest were subjected to in-gel trypsin digestion as described by Shevchenko et al. [5] with minor modifications as described previously by Kumar et al. [1].

Solution phase glycoprotein samples were subjected to proteolysis by trypsin through Filter Aided Sample Preparation (FASP) method using YM30/YM10 ultracentrifugal units (Millipore) as previously described [6] to obtain peptides for glycopeptide capture and gel free 2D LC–MALDI TOF/TOF approach. Tryptic peptides were lyophilized and stored in −80 °C prior to use.

### 3.6 Glycopeptide capture

Tryptic peptides obtained from the glycoprotein fraction (Con A Eluant) were dissolved in buffer containing 10 mM HEPES–NaOH (pH 7.5), 1 mM Cacl_2_, 1 mM MnCl_2_, 1 mM MgCl_2_ and subjected to Con A based lectin affinity chromatography as described by Kaji et al. [7] with minor modifications as described previously [1]. Since plant glycoproteins are known to posses α 1,3-linked core fucose we have utilized both PNGase A and PNGase F enzymes to deglycosylate the glycosylated peptides and to identify the potential N-glycosylation sites. Briefly, glycopeptide fractions were dried in vacuum and dissolved either in 50 mM Sodium phosphate buffer (pH 7.0) or in 50 mM Citrate phosphate buffer (pH 5.0) for PNGase F or PNGase A based deglycosylation respectively.

### 3.7 Database search strategy for protein identification

Tryptic peptides from 1D SDS PAGE, solution based samples and deglycosylation fractions were injected into Nano-LC system, fractionated and spotted onto PAC (Pre Anchored Chip) MALDI target plates. The plates were analyzed using Ultraflex III MALDI TOF/TOF mass spectrometer (Bruker Daltonics). Acquired mass spectra were analyzed and annotated using Flexanalysis software version 3.0 through WARP-LC (Workflow Administration by Result driven Processing – Bruker Daltonics) software tool. Protein identities were attained using Biotools version 3.2 equipped with in-house licensed Mascot server (version 2.3). Five different databases were employed to achieve protein identification, briefly NCBInr database (11/07/2015) containing 69,146,588 sequences (total) including 30,66,261 sequences from green plants (Viridiplantae), *G. arboreum* protein database containing 40,134 sequences downloaded from CottonGen website (*G. arboreum* (A2) genome BGI assembly v2.0 (annot v1)) (ftp://ftp.bioinfo.wsu.edu/species/Gossypium_arboreum/CGP-BGI_G.arboreum_Agenome/genes/G.arboreum_BGI-A2_v1.0_protein.fasta.gz), *G. raimondii* protein database containing 77,267 sequences downloaded from Cotton Gen website (*G. raimondii* (D5) JGI assembly v2.0 (annot v2.1)) (ftp://ftp.bioinfo.wsu.edu/species/Gossypium_raimondii/JGI_221_G.raimondii_Dgenome/genes/G.raimondii_JGI_221_v2.1.proteins.fasta.gz), *G. hirsutum* protein database containing 76,943 sequences downloaded from Cotton Gen website (ftp://ftp.bioinfo.wsu.edu/species/Gossypium_hirsutum/CGP-BGI_G.hirsutum_AD1genome/genes/BGI_Gossypium_hirsutum_v1.0.pep.gz) and *G. raimondii* protein database containing 40,976 sequences downloaded from CottonGen website (ftp://ftp.bioinfo.wsu.edu/species/Gossypium_raimondii/CGP-BGI_G.raimondii_Dgenome/genes/). Database search parameters used were: Taxonomy – Viridiplantae (Green plants), Enzyme – Semi trypsin/trypsin, Fixed modifications: Carbamidomethylation of Cysteine (C), Variable Modifications: Oxidation of Methionine (M), Carbamylation of Lysine (K) and N-terminus of protein, Protein mass was unrestricted, Missed cleavage was set to 1, MS tolerance of ±100 ppm and MS/MS tolerance of ±0.75 Da. The protein identification parameters were as follows: Significance threshold was set to achieve *p*<0.02, Expectancy cut off was set to 0.05, Individual ion score >45 was only considered for identification. These parameters led to a FDR value <1% in both the above mentioned database search strategies. The database search strategy for deglycosylated peptide identification is the same as mentioned above with minor additions: variable modifications included deamidation of asparagine (N), the peptide is considered as formerly glycosylated only if the deamidated asparagine (N) was followed by X-S/T (any amino acid except proline–serine/threonine). Also only those peptides that were observed in three replicate sample injections are reported as formerly glycosylated peptides in the current study.

### 3.8 Data analysis using Scaffold and bioinformatic tools

Mascot DAT files were extracted from individual samples and exported into Scaffold software (version 4.4.1.1). Spectral files were subjected to protein identification through the above mentioned databases. Data analysis parameters were set as follows: FDR <1%, Minimum number of unique peptides: 1, Protein identification probability: >95%. Analysis of the dataset with additional databases retrieved 21 N-linked glycosylation sites and 18 additional glycoproteins that were not reported in our previous study (Table 1, Table 2 and Supplementary Table 1). Our analysis also revealed that *G. hirsutum* (AD) harbors protein sequences specific to its parental species viz., *G. arboreum* (A) and *G. raimondii* (D). Identified proteins were exported to BLAST2GO platform (version 2.0) (http://www.blast2go.com/b2ghome) [8] for Gene Ontology (GO) annotation, protein motif prediction and pathway mapping. Potential N-terminal signal peptides were predicted using SignalP 4.0 server (http://www.cbs.dtu.dk/services/SignalP) [9], Integral transmembrane domain prediction using TMHMM-2.0(http://www.cbs.dtu.dk/services/TMHMM), potential N-Linked glycosylation site was predicted using Net N-Glyc (http://www.cbs.dtu.dk/services/NetNGlyc), Glycosylphosphatidyl inositol (GPI)-anchored proteins were predicted using Big-PI plant predictor tool (http://www.mendel.imp.ac.at/gpi/plant_server.html) [10]. Proteins were classified into different carbohydrate active enzyme (CAZyme) families through CAZYmes Analysis Toolkit (CAT) (http://www.mothra.ornl.gov) [11]. Leucine rich repeat sequence prediction was carried out using LLR finder tool (http://www.lrrfinder.com). Peroxidases were analyzed and classified using Peroxibase database (http://www.peroxibase.toulouse.inra.fr/peroxiscan.php) [12].

## Conflict of interest

The authors declare they have no conflict of interest.

## Figures and Tables

**Table 1:** Glycosylation site assignment using MALDI TOF/TOF (Additional glycosylation sites exclusively reported in the current study).

**Sl no.**	**Accession no.**	**Protein ID – [Source]**	**Observed Mole Wt**	**Sequence coverage %**	**Peptides sequenced by MS/MS+modifications**	**MOWSEscore**

1	Gorai.008G010600.1	PREDICTED: desiccation-related protein PCC13-62-like [*Gossypium raimondii*]	2669.3126	12	R.TLLNDVI**N(+.98)**STVQPYTFTAAELS**N(+.98)**R.T+2 Deamidated (NQ)	224
	Cotton_A_34455_BGI-A2_v1.0				
	CotAD_12989				
	Cotton_D_gene_10038147*	Predicted protein [*Populus trichocarpa*] Dessication related protein				
2	Cotton_A_24352_BGI-A2_v1.0	PREDICTED: PI-PLC X domain-containing protein At5g67130 [*Gossypium raimondii*]	2949.3709	12	R.SLILQNYFPSNP**N(+.98)**ETAACAENSAPLVK.M+Deamidated (NQ)	209
	CotAD_35067				
	Gorai.007G214400.8					
3	CotAD_16513	Proline--tRNA ligase [*Gossypium arboreum*]	2197.959	4	K.LSC**N(+.98)**SSSSSSEAGFSQATSFR.M+Deamidated (NQ)	190
	Cotton_A_23586_BGI-A2_v1.0				
	Gorai.001G140300.2					
4	Gorai.010G096500.1	Pathogenesis-related PR-1 type [*Gossypium arboreum*]	1825.9076	7	K.LTDNPADELVAVLNA**N**(+.98)R.T+Deamidated (NQ)	119
	CotAD_53847					
	Cotton_D_gene_10026388*					
5	Cotton_A_16611_BGI-A2_v1.0	Monocopper oxidase-like protein SKU5 [*Gossypium arboreum*]	2044.058	7	K.FPGPTI**N(+.98)**STTNNNVVVNVR.N+Deamidated (NQ)	119
	CotAD_19756					
	Cotton_D_gene_10015446					
6	Gorai.009G246700.1	PREDICTED: protein disulfide-isomerase-like [*Gossypium raimondii*]	1396.668	4	R.STSG**N(+.98)**ITPYEGNR.T+Deamidated (NQ)	88
	Cotton_A_16089_BGI-A2_v1.0					
	CotAD_73953					
	Cotton_D_gene_10007690*$					
7	Gorai.013G030200.1	PREDICTED: probable receptor protein kinase TMK1 [*Gossypium raimondii*]	1692.7943	1	R.L**N(+.98)**GTIEVIQ**N(+.98)**MSSLR.E+2 Deamidated (NQ); Oxidation (M)	47
	Cotton_A_00858_BGI-A2_v1.0					
	CotAD_24219					
	Cotton_D_gene_10024539					
8	Cotton_A_24352_BGI-A2_v1.0	PREDICTED: PI-PLC X domain-containing protein At5g67130 [*Gossypium raimondii*]	2409.0506	7	Q.NYFPTNP**N(+.98)**ETAACAENSAPLVK.M+Deamidated (NQ)	152
	CotAD_35067					
	Gorai.007G214400.1					
9	Gorai.004G201700.1	PREDICTED: protein YLS3-like [*Gossypium raimondii*]	2572.1033	10	S.CYPYLN**N(+.98)**ATAKPEDDCCNPIR.Q+Deamidated (NQ)	47
	Cotton_A_36086_BGI-A2_v1.0					
	CotAD_48188					
10	Gorai.007G021100.2	PREDICTED: lipid transfer-like protein VAS [*Gossypium raimondii*]	1560.7793	12	F.ASL**N(+.98)**ISTAQAL**N(+.98)**VTR.E+2 Deamidated (NQ)	135
	CotAD_02614					
11	Gorai.008G216800.1	PREDICTED: acid beta-fructofuranosidase-like [*Gossypium raimondii*]	2176.928	9	R.PL**N(+.98)**TSDGSLETYFCADETR.S+Deamidated (NQ)	50
	CotAD_31849					
12	Cotton_A_11565_BGI-A2_v1.0	PREDICTED: GDSL esterase/lipase At5g14450-like [*Gossypium raimondii*]	1686.8147	9	R.RP**N(+.98)**ETIYEYGISPF.G+Deamidated (NQ)	103
	Gorai.001G266600.1					
	CotAD_07905					
13	Gorai.006G117500.1	PREDICTED: protein STRICTOSIDINE SYNTHASE-LIKE 3-like [*Gossypium raimondii*]	1061.3817	3	K.DFAYTSS**N(+.98)**R.S+Deamidated (NQ)	45
	CotAD_27689					
14	Cotton_A_36266_BGI-A2_v1.0	Hydantoin utilization C [*Gossypium arboreum*] Isoform of S.NO 25	1622.6905	3	K.TTVDGQ**N(+.98)**ISFLEAAR.S+Deamidated (NQ)	51
	CotAD_68215					
15	Gorai.003G061600.1	PREDICTED: lysM domain receptor-like kinase 3 isoform X1 [*Gossypium raimondii*]	1288.691	1	K.NYAT**N(+.98)**TTFTVR.S+Deamidated (NQ)	47
	CotAD_65258					
	Cotton_A_27440_BGI-A2_v1.0					
16	CotAD_16513	Proline--tRNA ligase [*Gossypium arboreum*]	3367.4499	6	S.GV**N(+.98)**YSSSVGMKLSC**N(+.98)**SSSSSSEAGFSQATSFR.M+Carbamyl (N-term); 2 Deamidated (NQ); Oxidation (M)	150
	Cotton_A_23586_BGI-A2_v1.0					
	Gorai.001G140300.3					
17	CotAD_72223	PREDICTED: beta-xylosidase/alpha-L-arabinofuranosidase 2 [*Gossypium raimondii*]	1502.7566	4	T.AASF**N(+.98)**TSLYETIGK.V+Deamidated (NQ)	50
	Cotton_A_27349_BGI-A2_v1.0					
	Gorai.005G218000.2					


**Table 2:** Additional protein identified in the current study using *Gossypium* species specific protein sequence databases.

**Sl no.**	**Accession no.**	**Protein ID – [Source]**	**Mole Wt (kDa)**	**Observedmass(M+H)^+^**	**Peptides sequenced by MS/MS**	**MOWSE score**

1	Gorai.002G207900.1	Predicted:Separase isoform X1 [*Gossypium raimondii*] Pepridase_C50	215.624	1140.65	S.ADHLSEDVVR.D	58
2	CotAD_40266 Gorai.004G002500.1	Predicted:cucumisin-like [*Gossypium raimondii*] Peptidase_S8_3	144.236	2049.08	K.**L**AGNNTS**C**PKNSTK**M**LPR.D+Carbamyl (L), Carbamidomethyl (C), Oxidation (M)	54
3	CotAD_03361 Gorai.009G100400.1	Predicted: heat shock cognate 70 kDa protein 2 like [*Gossypium raimondii*]	71.222	1568.80	K.**Q**FAAEEISSMVLMK.M+Ammonia-loss (Q)	45
4	Cotton_A_01713	Aldehyde oxidase 4-like protein [*Gossypium arboreum*]	145.122	2283.38	K.QMTAYGLGLVK**C**GGTQDLLEK.V+Carbamidomethyl (C)	57
5	Cotton_A_01770	Predicted:Cysteine proteinase inhibitor like [*Gosyypium raimondii*]	11.388	2357.10	M.**A**TLGGISQVDGSANSLEIENLAR.F+Acetyl (A)	62
6	Cotton_A_01738	Predicted:Auxin response factor 4-like isoform X1 [*Gosyypium raimondii*]	84.086	1429.76	P.RASHAEFVIPFR.K	65
7	Cotton_A_01780	Predicted:crt homolog 1 isoform X3 [*Gosyypium raimondii*] CRT-Chlorouine resistance transporter	47.582	1165.45	P.VGRSEGGD**C**TK.K+Carbamidomethyl (C)	58
8	Cotton_A_01799	Ribonuclease I [*Gossypium arboreum*]	104.392	1026.56	V.TGGSGSIYER.F	63
9	CotAD_36535 Cotton_A_04276	Protein disulfide isomerase-like 1-4 [*Gossypium arboreum*]	65.760	1619.74	Y.FFVDGKHKPYPGAR.N	59
10	CotAD_73953	Predicted:Protein disulfide-isomerase-like [*Gossypium raimondii*]	52.851	1748.81	I.EDATANDIVGENFDVR.G	90
11	CotAD_38843	PREDICTED: heat shock cognate 70 kDa protein 2-like isoform X1 [*Gossypium raimondii*]	70.793	1740.73	K.ETAEAYLGSTVKNGVVT.V	90
12	CotAD_45862	Fructokinase [*Gossypium hirsutum*]	34.965	1616.77	K.**Q**NGVSGDGILFDQGAR.T+Ammonia-loss (Q)	72
13	CotAD_44107 Gorai.002G182000.1	Predicted:nuclear transport factor 2 [*Gossypium raimondii*]	13.373	1705.87	K.AFVDHYYSTFDANR.A	42
14	CotAD_24219 Gorai.013G030200.1	PREDICTED: probable receptor protein kinase TMK1 [*Gossypium raimondii*]	102.438	1692.79	R.L**N**GTIEVIQ**NM**SSLR.E+2 Deamidated (N); Oxidation (M) (Glycopeptide)	47
15	CotAD_02614 Gorai.007G021100.2	PREDICTED: lipid transfer-like protein VAS [*Gossypium raimondii*]	15.572	1560.77	F.ASL**N**ISTAQAL**N**VTR.E+2 Deamidated (NQ) (Glycopeptide)	135
16	CotAD_27689 Gorai.006G117500.1	PREDICTED: protein STRICTOSIDINE SYNTHASE-LIKE 3-like [*Gossypium raimondii*]	43.827	1061.38	K.DFAYTSS**N**R.S+Deamidated (N) (Glycopeptide)	45
17	CotAD_68215 Cotton_A_36266	Hydantoin utilization C [*Gossypium arboreum*]	51.739	1622.69	K.TTVDGQ**N**ISFLEAAR.S+Deamidated (N) (Glycopeptide)	51
18	CotAD_65258 Gorai.003G061600.1	PREDICTED: lysM domain receptor-like kinase 3 isoform X1 [*Gossypium raimondii*]	73.564	1288.69	K.NYAT**N**TTFTVR.S+Deamidated (N) (Glycopeptide)	47

