# Supplementary material for: Delineating the glycoproteome of elongating cotton fiber cells
Source: Data Brief. 2015 Oct 26;5:717–25. doi: 10.1016/j.dib.2015.10.015 (PMC4652025; doi:10.1016/j.dib.2015.10.015)
Supplement: Supplementary file 2 — Supplementary material [file mmc2.doc]

**Conflict of interest**

The authors declare they have no conflict of interest
